# PM2.5-induced oxidative stress upregulates PLA2R expression in the lung and is involved in the pathogenesis of membranous nephropathy through extracellular vesicles

**DOI:** 10.3389/fphar.2024.1516111

**Published:** 2024-12-18

**Authors:** Wang Zhang, Jiating Chen, Ye Yuan, Jiao Luo, Zhanmei Zhou, Guobao Wang

**Affiliations:** ^1^ Renal Division, State Key Laboratory of Organ Failure Research, National Clinical Research Center for Kidney Disease, Nanfang Hospital, Southern Medical University, Guangzhou, China; ^2^ Department of Nephrology, The Second Affiliated Hospital of Shenzhen University, Shenzhen, China

**Keywords:** oxidative stress, membranous nephropathy, extracellular vesicles, PM 2.5, PLA2R

## Abstract

**Background:**

Particulate matter (PM2.5) has been implicated in the development of membranous nephropathy (MN), but the underlying mechanism has yet to be fully understood. Oxidative stress is an essential factor of PM2.5-related toxicity and plays a significant role in the exposure of target antigenic epitopes in MN. This study aims to explore the pathogenic effects of PM2.5 in facilitating the crosstalk between the lung and kidney in MN.

**Method:**

We examined oxidative stress indicators and the circulating levels of extracellular vesicles (EVs) in patients diagnosed with MN. Additionally, we assessed the expression of M-type phospholipase A2 receptor (PLA2R) in human lung tissue *ex vivo*. To verify the impact of PM2.5 on PLA2R expression in the lung and the kidney, we stimulated human bronchial epithelial cells (Beas-2B) with lipopolysaccharide (LPS) or PM2.5. We then treated podocytes *in vitro* with the supernatants from PM2.5-exposed Beas-2B cells, intervening with GW4869, an inhibitor of EV release, to explore the role of EV-mediated cell-cell interactions.

**Results:**

We found that elevated serum markers of oxidative stress and increased levels of PLA2R + EVs correlated positively with anti-PLA2R antibody levels in the serum of patients with idiopathic MN (IMN). Notably, PLA2R expression was significantly higher in the lung tissue of smokers, suggesting a possible link between PLA2R and oxidative stress. *In vitro* experiments demonstrated that PLA2R expression in Beas-2B cells was upregulated upon stimulation with LPS and PM2.5, an effect that could be reversed by the antioxidant glutathione (GSH). Furthermore, the supernatants from PM2.5-exposed Beas-2B cells were found to induce PLA2R overexpression and injury in podocytes, with this effect being mitigated by GW4869, an inhibitor of EVs release.

**Conclusion:**

Our study contributes new knowledge to the understanding of how environmental pollutants, such as PM2.5, cause kidney damage through oxidative stress and EV-mediated signaling. The findings pave the way for further research into therapeutic strategies targeting oxidative stress and EVs, which could potentially improve patient outcomes of MN, particularly in high-risk populations like smokers and those exposed to air pollution.

## 1 Introduction

Membranous nephropathy (MN) is a common cause of nephrotic syndrome in adults, characterized by immune complex deposits on the subepithelial side of the glomerular basement membrane (GBM). Idiopathic MN (IMN), which comprises 70%–80% of cases, lacks determined secondary causes. In 2009, secretory M-type phospholipase A2 receptor (PLA2R1) was identified as the primary autoantigen, present in 80% of IMN cases (Beck et al., 2009). Detection of anti-PLA2R1 antibodies (aPLA2R1ab) in peripheral blood has become pivotal in clinical diagnosis, prognosis prediction, and treatment guidance (van de Logt et al., 2019). However, the pathogenesis of PLA2R-associated MN has yet to be elucidated fully.

In recent years, a remarkable increase in the prevalence of MN in China has been witnessed. An epidemiological study conducted in our cent (Xu et al., 2016) re revealed that for every 10 mg/m^3^ rise in fine particulate matter less than 2.5 μm (PM2.5) concentration over 70 mg/m^3^, there is a 14% higher likelihood of MN. Similar findings were reported by Zhang et al., indicating that exposure to PM2.5 was associated with an increased incidence of MN in northern China (Li et al., 2018). However, the precise involvement of PM2.5 in the pathogenesis of MN remains unclear.

Experimental studies have established that oxidative stress plays a crucial role in PLA2R antigenic epitope exposure, which triggers the production of pathogenic antibodies (Liu et al., 2019; Ronco and Debiec, 2020). PM2.5, one of the most common causes of oxidative stress (Li et al., 2018; Xu et al., 2016), induces lung inflammatory responses. Based on this, we hypothesized that lung exposure to oxidative stress prompts the production of specific pathogenic antibodies implicated in MN.

This study aims to assess the effect of oxidative stress on PLA2R expression in the lung and investigate the mechanism of lung-kidney interaction under PM2.5 exposure.

## 2 Materials and methods

### 2.1 Subjects

To analyze plasma extracellular vesicles (EVs), we evaluated eight patients with IMN who tested positive for serum anti-PLA2R antibodies at our hospital between January 2021 and December 2022. Four patients diagnosed with minimal change disease (MCD), admitted during the same period, served as controls. Exclusion criteria encompassed patients with diabetes, cardiovascular disease, hepatic disease, a history of dust exposure or respiratory infections, or those receiving glucocorticoids and/or immunosuppressive agents within 3 months prior to enrollment.

Additionally, five patients who underwent lobectomy for lung cancer between November 2021 and February 2022 were categorized into smoking (SM) and non-smoking (NSM) groups. In these cases, normal lung tissue adjacent to cancer lesions was utilized for PLA2R detection.

All study participants provided informed consent. The experiment adhered to ethical standards for human research and received approval from the Medical Ethics Committee of Nanfang Hospital.

### 2.2 Cell culture and treatment

#### 2.2.1 Beas-2B

The human bronchial epithelial cell line (Beas-2B) was maintained in Dulbecco’s modified Eagle medium (DMEM) supplemented with 10% fetal bovine serum (FBS) and 1% penicillin and streptomycin. The culture medium was then collected and centrifugated at 6,000 rpm for 5 min at 4°C to obtain the supernatants. LPS (1 ug/mL) was given for stimulation for 24 h, and when needed, Beas-2B cells were pretreated with GSH (1 mmol/L) for 4 h to alleviate oxidative stress. RM8785 (50ug/mL) was given for 24 h. For EVs blockade, the cells were pretreated with GW4869 (20 μM) for 30 min prior to RM8785 exposure.

#### 2.2.2 Podocyte

Conditionally immortalized human podocytes (HPCs) were cultured as described previously (Ni et al., 2012). The cells were cultivated with DMEM high glucose culture solution incorporating 10% fetal bovine serum and 1% penicillin/streptomycin in an incubator with 5% CO2 and saturated humidity at 37°C. The experiments were conducted on cells in the logarithmic growth phase. The podocytes were treated with conditioned medium or extracellular vesicles from Beas-2B. The injury of podocyte was assessed following treatment for 24 h.

### 2.3 Isolation and characterizing of EVs

#### 2.3.1 Plasma EVs isolation

All subjects signed an informed consent prior to blood collection. Venous blood samples (4 mL each) were obtained from eight IMN and four MCD patients using Vacutainer Plus tubes containing EDTA. Plasma was isolated by centrifugation at 2,000 *g* for 10 min, followed by 100,00× g for 30 min. The plasma was then transferred to a new tube, and EVs were pelleted by ultracentrifugation at 100,000 × g for 120 min. All steps were performed at 4°C. The EVs pellets were washed with 1 mL of 0.22 μM filtered Dulbecco’s PBS (−/−) and centrifuged again at 1,000,00 × g for 60 min at 4°C and resuspended in PBS before storage at −80°C (Coumans et al., 2017; Muller et al., 2014).

#### 2.3.2 Lung tissue EVs isolation

EVs were extracted from the lung tissue according to a modified protocol described by Crescitelli et al. (Crescitelli et al., 2021). Initially, lung tissues were weighed and immersed in RPMI-1640 medium within Petri dishes, then sliced into approximately 2 mm × 2 mm × 2 mm pieces. These tissue segments were subsequently incubated for 30 min at 37°C in RPMI-1640 medium supplemented with collagenase D (2 mg/mL) and DNase I (40 U/mL). Following incubation, the EV-containing medium was filtered through a 70 μm cell strainer to remove tissue fragments. To further purify the sample by eliminating cells and debris, it underwent centrifugation at 500 *g* for 10 min, followed by 2,000× g for 20 min, repeated twice to eliminate apoptotic bodies and larger EVs. The resulting supernatant was transferred to a fresh tube, and exosomes were pelleted at 1,180,00× g for 150 min at 4°C. Finally, the supernatant was discarded, and the pellet containing freshly isolated tissue exosomes was resuspended in PBS and stored at −80°C.

#### 2.3.3 Conditioned media EVs isolation

Briefly, after removing cell debris by centrifugation at 300 *g* for 5 min, 2,000 g for 20 min, and 10,000 g for 30 min, respectively, the supernatant was ultra-centrifuged at 110,000 g for 1 h, and all steps were performed at 4°C. The final pellets of exosomes were resuspended and stored at −80°C.

#### 2.3.4 Nanoparticle tracking analysis (NTA)

The vesicle suspensions with concentrations between 1 × 10^7^/mL and 1 × 10^9^/mL were examined using the Zeta View PMX 110 (Particle Metrix, Meerbusch, Germany) equipped with a 405 nm laser to determine the particle concentration, size, or distribution of exosomes.

#### 2.3.5 Transmission electron microscopy (TEM)

Freshly isolated EVs were placed on a carbon-coated copper grid and incubated at room temperature for 5 min. Then, the EVs were immersed in 2% (v/v) uranyl acetate solution for 1 min. Excess fluid was removed using filter paper. The EVs were observed and imaged under a transmission electron microscope at an accelerating voltage of 80 kV.

#### 2.3.6 Immunogold labelling of EVs

Immunogold labelling of EVs was conducted following the protocol outlined by Thery et al. (2006). In brief, purified and concentrated EVs stored at −80°C were thawed and mixed with an equal volume of 4% paraformaldehyde (PFA) fix solution. A 5 μL aliquot of the resuspended pellets was then deposited onto Formvar-carbon-coated electron microscopy grids. Grids were then transferred to drops of PBS on Parafilm and repeatedly washed in PBS and PBS containing 50 mM glycine. Grids were next incubated in a drop of 5% bovine serum albumin (BSA) blocking buffer for 10 min.

After blocking, grids were incubated in a 5 μL drop of primary anti-PLA2R antibody (ab211573, Abcam) diluted 1:20 (v/v) in the blocking buffer for 40 min. Grids were then incubated in 5 μL drops of secondary antibody conjugated with protein A-gold, diluted in blocking buffer, for 20 min. Following secondary Ab incubation, grids were repeatedly washed and immersed in 50 μL drops of 1% glutaraldehyde for 5 min. Finally, exosomes were treated with a 2% uranyl acetate solution for 1 min before observation under an 80 kV electron microscope.

#### 2.3.7 Nanoscale flow cytometry

Isolated EVs were captured on aldehyde sulfate-coated latex beads. Blocking of the beads with 0.5% w/v BSA was performed prior to capture. The captured exosomes were detected with PE-Cy^TM^7 mouse anti-human CD63Ab (561982, BD Bioscience) and FITC mouse anti-human PLA2R Ab (NBP2-50248F, Novus Biologicals).

### 2.4 Western blot analysis

Protein expression was analyzed using Western blot analysis. The primary antibodies used were described as follows. Abs specific for the PLA2R (ab211490, Abcam). Abs specific for EVs markers: TSG101 (ab228013, Abcam), CD63 (ab68418, Abcam). Abs specific for oxidative stress: NOX2 (ab129068, Abcam), NOX4 (BA2813, Boster Biological Technology). Abs specific for podocyte: Nephrin (ab58968, Abcam), ZO-1 (40-2200, Invitrogen). Abs specific for the housekeeping protein GAPDH (RM 2002, Beijing Ray Antibody Biotech).

### 2.5 Histology and immunohistochemical staining

A routine procedure prepared paraffin-embedded human lung tissue sections. Anti-PLA2R antibody (ab211573, Abcam) was used as a primary antibody. Hematoxylin-eosin and Masson’s trichrome and immunohistochemical staining were performed using routine protocol.

### 2.6 Immunofluorescence staining

Human lung tissue and Beas-2B cells cultured on coverslips were incubated with primary antibodies: Anti-PLA2R antibody (ab211490, Abcam), Anti-RAGE antibody (ab216329, Abcam), Anti-Prosurfactant Protein C/SFTPC antibody (ab90716, Abcam). After washing, the slides were incubated with Alexa Fluor^®^ 488 conjugated goat anti-mouse (ab150113, Abcam) or Alexa Fluor^®^ 568 goat anti-rabbit IgG (ab175471, Abcam). Images were taken by fluorescence microscopy.

### 2.7 Statistical analysis

The data were reported as means ± standard deviation (SD) with the sample size. All statistical analyses were conducted using GraphPad Prism 8 software. All the sample sizes (n) were shown in figure legends. Comparisons between groups were made using one-way ANOVA, followed by the Student-Newman-Kuels test or Dunnett’s T3 procedure. A p-value <0.05 was considered statistically significant.

## 3 Results

### 3.1 Oxidative stress associated with PLA2R overexpression

**FIGURE 1:**
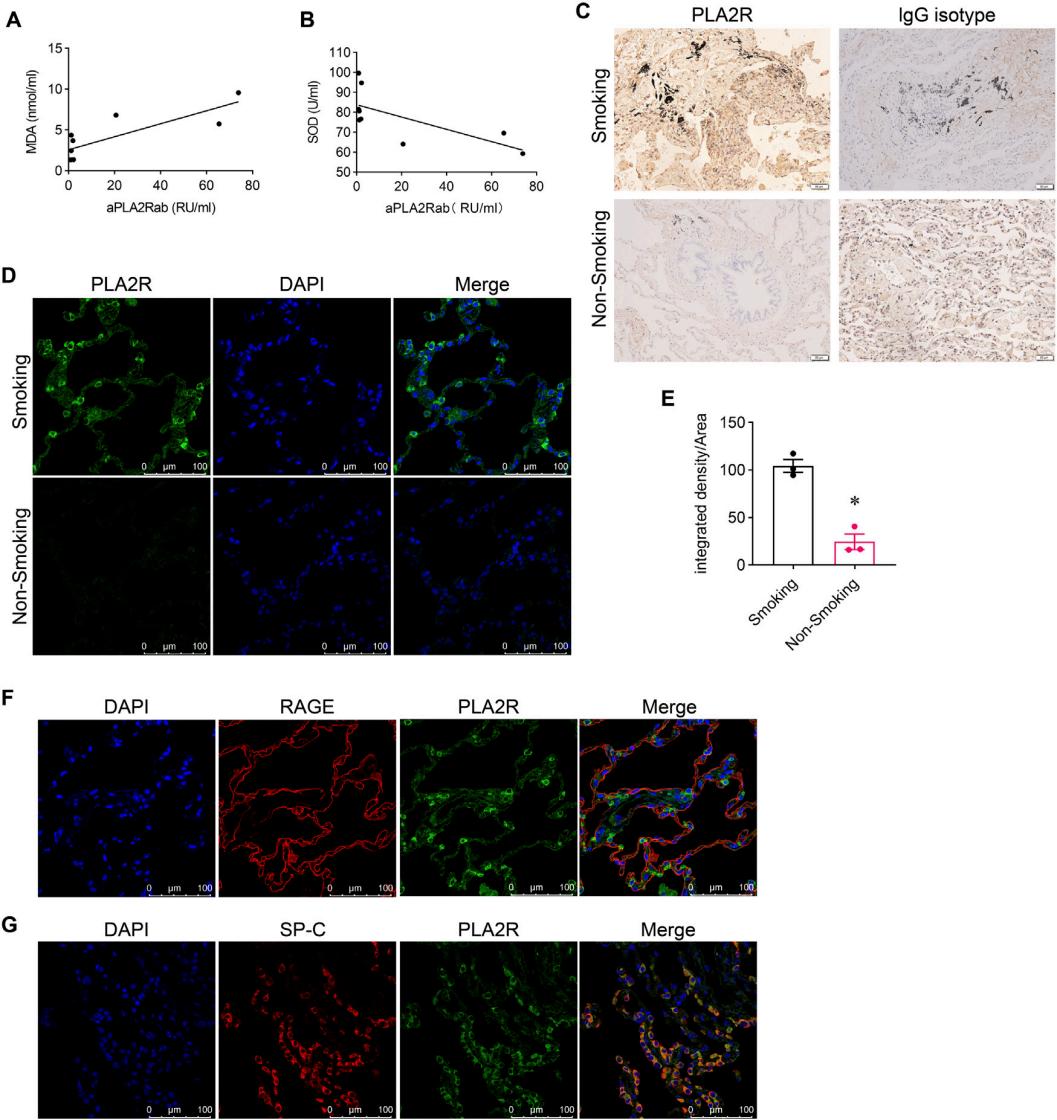
Association of PLA2R overexpression with oxidative stress. **(A)** Malondialdehyde (MDA), a marker of oxidative stress, shows a positive relationship with serum levels of anti-PLA2R antibodies. **(B)** Superoxide Dismutase (SOD), an antioxidant enzyme, demonstrates a negative correlation with serum anti-PLA2R antibody levels. **(C)** Immunohistochemistry indicates PLA2R expression in lung tissues of both smokers and non-smokers. Scale bar, 50 μm. **(D, E)** Representative and quantitative data from immunofluorescence analysis reveal that PLA2R expression in the lung tissues of smokers is significantly higher than in non-smokers. Scale bar = 100 μm *p < 0.05. **(F, G)** Immunofluorescence micrographs demonstrate the co-localization of PLA2R with RAGE (receptor for advanced glycation end products, a marker for Type Ⅰ alveolar epithelium) and SP-C (surfactant protein C, a marker for Type Ⅱ alveolar epithelium) in the lung tissue of smokers. Scale bar = 100 μm.

We measured oxidative stress indicators (MDA, SOD) and anti-PLA2R antibody (aPLA2Rab) levels in the serum of patients with IMN. The levels of aPLA2Rab were 30.21 ± 42.63 RU/mL, MDA was 5.18 ± 4.42 nmol/mL, and SOD was 77.28 ± 11.01 U/mL. We found a positive correlation between aPLA2Rab and MDA (Figure 1A) and a negative correlation with SOD levels (Figure 1B).

To explore the relationship between oxidative stress and PLA2R expression in the lung, we initially compared PLA2R expression in lung tissues from smokers (SM) and non-smokers (NSM). We found markedly stronger PLA2R expression in smokers’ lungs than in non-smokers using immunohistochemistry (Figure 1C). Immunofluorescence further demonstrated significant PLA2R expression in smokers’ lung tissues (Figures 1D, E), predominantly co-localizing with SP-C, an alveolar type II epithelium marker (Figure 1G), instead of RAGE (a marker for Type I alveolar epithelium) (Figure 1F).

### 3.2 PM2.5 induces oxidative stress and overexpression of PLA2R in bronchial epithelium

**FIGURE 2:**
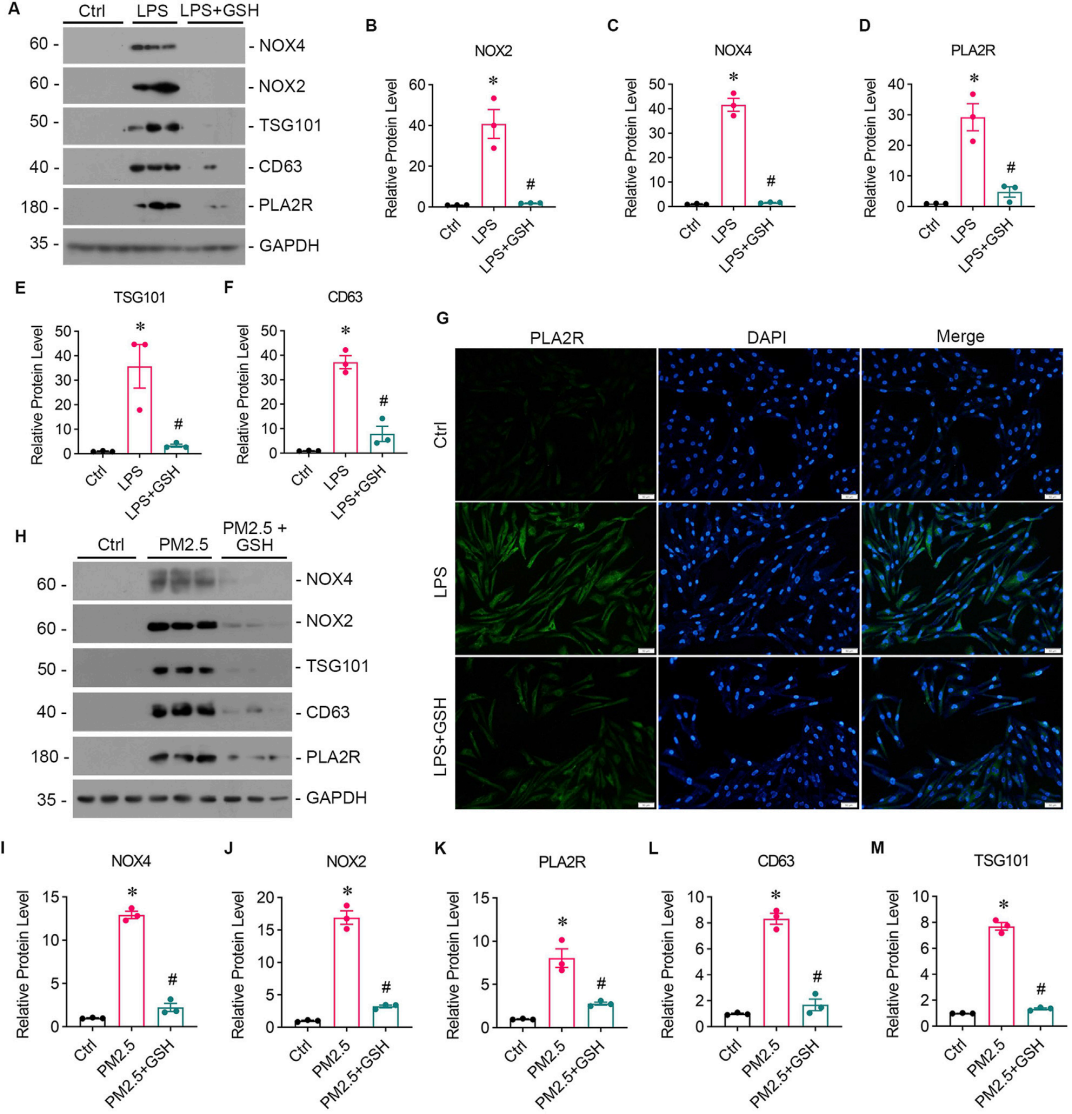
PM2.5 induces oxidative stress and PLA2R overexpression in bronchial epithelium. **(A–F)** Levels of oxidative stress, PLA2R expression, and EVs production in Beas-2B cells stimulated by LPS, with or without GSH intervention. Western blot **(A)** and quantitative data **(B–F)** are presented. **(B, C)** NOX2 and NOX4 are oxidative stress markers from the NADPH oxidase family. **(D)** PLA2R expression levels. **(E, F)** TSG101 and CD63 are markers for EVs. All are significantly upregulated by LPS, while GSH effectively reverses this upregulation. (*p < 0.05 versus control; #P < 0.05 versus LPS). **(G)** Representative micrographs show immunofluorescence staining of PLA2R in Beas-2B cells treated with LPS, with or without GSH. Scale bar = 100 μm. **(H–M)** Levels of oxidative stress, PLA2R expression, and EVs production in Beas-2B cells stimulated by RM8785, with or without GSH intervention. Representative Western blot **(H)** and quantitative data **(I–M)** are provided. The expression levels of NOX4 **(I)**, NOX2 **(J)**, PLA2R **(K)**, CD63 **(L)**, and TSG101 **(M)** are all significantly upregulated by RM8785, while GSH effectively reverses this upregulation. (*p < 0.05 versus control; #P < 0.05 versus PM2.5).

To investigate the impact of oxidative stress on PLA2R expression, we stimulated bronchial epithelial cells (Beas-2B) with LPS. Western blotting showed upregulated expression of NOX2 (Figures 2A, B), NOX4 (Figures 2A, C), and PLA2R (Figures 2A, D). Immunofluorescence also indicated heightened PLA2R expression with LPS stimulation (Figure 2G).

Long-term exposure to PM2.5 is known to cause oxidative stress, a significant contributor to pulmonary epithelial damage. We then investigated whether PM2.5 could upregulate PLA2R expression in bronchial epithelial cells using Reference Material (RM) 8785, an air particulate matter on filter media, incubated with Beas-2B cells. In response to RM8785, there was a significant increase in PLA2R expression (Figures 2H, K) and oxidative stress (Figures 2H–J) levels in Beas-2B cells. Furthermore, the overexpression of PLA2R and oxidative stress indicators were reduced mainly by antioxidant GSH (Figures 2A, G, H). These findings suggest that PM2.5 induces the overexpression of PLA2R in pulmonary epithelial cells through oxidative stress.

### 3.3 Podocyte injury occurs as a result of oxidative stress damage in bronchial epithelial cell

**FIGURE 3:**
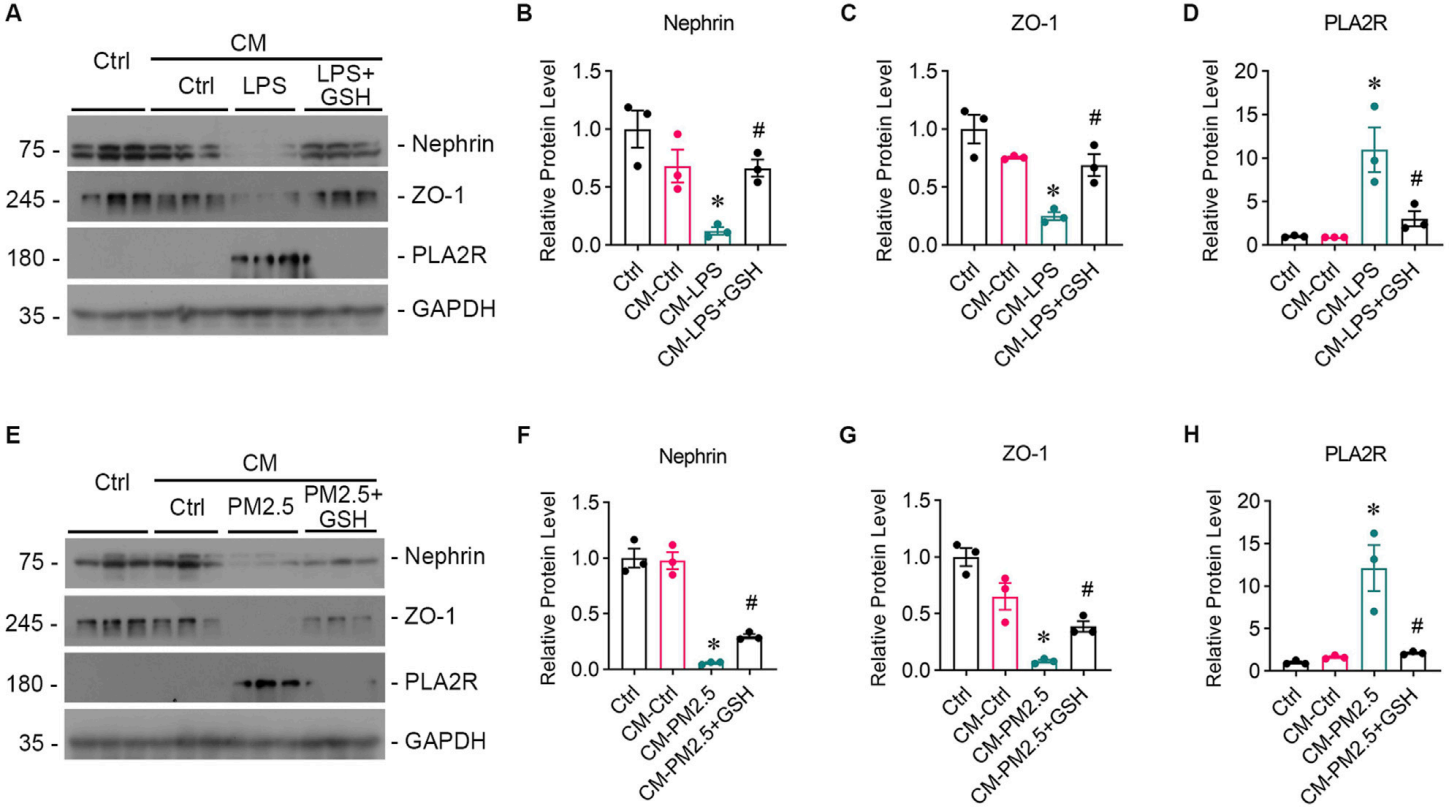
Podocyte injury induced by oxidative stress in bronchial epithelial cells. **(A)** Representative images of Western blot analyses show Nephrin, ZO-1, and PLA2R protein expression levels in podocytes exposed to supernatants from LPS-stimulated Beas-2B cells, with or without GSH intervention. CM-LPS: conditioned media from Beas-2B cells stimulated by LPS. CM-LPS + GSH: conditioned media from Beas-2B cells stimulated by LPS and pretreated with GSH. **(B, C)** The expression levels of Nephrin and ZO-1, both markers of podocytes, were downregulated by CM-LPS and were partially reversed by CM-LPS + GSH. **(D)** CM-LPS significantly elevated the expression of PLA2R in podocytes. (*p < 0.05 versus CM-Ctrl; #*p* < 0.05 versus CM-LPS). **(E)** Western blot images depict Nephrin, ZO-1, and PLA2R levels in podocytes exposed to supernatants from RM8785-stimulated Beas-2B cells, with or without GSH intervention. CM-PM2.5: conditioned media from Beas-2B cells stimulated by RM8785. CM-LPS + GSH: conditioned media from Beas-2B cells stimulated by RM8785 and pretreated with GSH. **(F, G)** The downregulation of Nephrin and ZO-1 by CM-PM2.5 were partially restored by CM-PM2.5+GSH. **(H)** PLA2R expression in podocytes was also increased by CM-PM2.5. (*p < 0.05 versus CM-Ctrl; #*p* < 0.05 versus CM-PM2.5).

To study whether oxidative stress-induced Beas-2B cells affected podocytes, Beas-2B cells were exposed to LPS *in vitro*, and the supernatant was collected. Then, podocytes were treated with the supernatants from LPS-exposed Beas-2B cells (referred to as CM-LPS), with the intervention of GSH (CM-LPS + GSH) or PBS-exposed cells (CM-Ctr) for 24 h, and podocyte markers were assessed (Figure 3A). The results showed a significant reduction in the expression of nephrin (Figures 3A, B) and ZO-1 (Figures 3A, C) caused by CM-LPS, indicating apparent podocyte injury. Additionally, the expression of PLA2R in podocytes was increased by supernatants from LPS-exposed Beas-2B cells (Figure3A, D). Furthermore, we collected the supernatants from Beas-2B cells stimulated by RM8785 (labelled as CM-PM2.5), with GSH intervention (CM- PM2.5+GSH) or from PBS-exposed cells (CM-Ctr) and treated podocytes to them. Exposure to CM-PM2.5 resulted in decreased expression of nephrin (Figures 3E, F) and ZO-1 (Figures 3E, G), along with increased expression of PLA2R (Figures 3E, H). These findings indicate that PM2.5-induced oxidative stress in lung epithelial cells can contribute to podocyte injury.

### 3.4 PM2.5 exposure promotes EVs secretion

**FIGURE 4:**
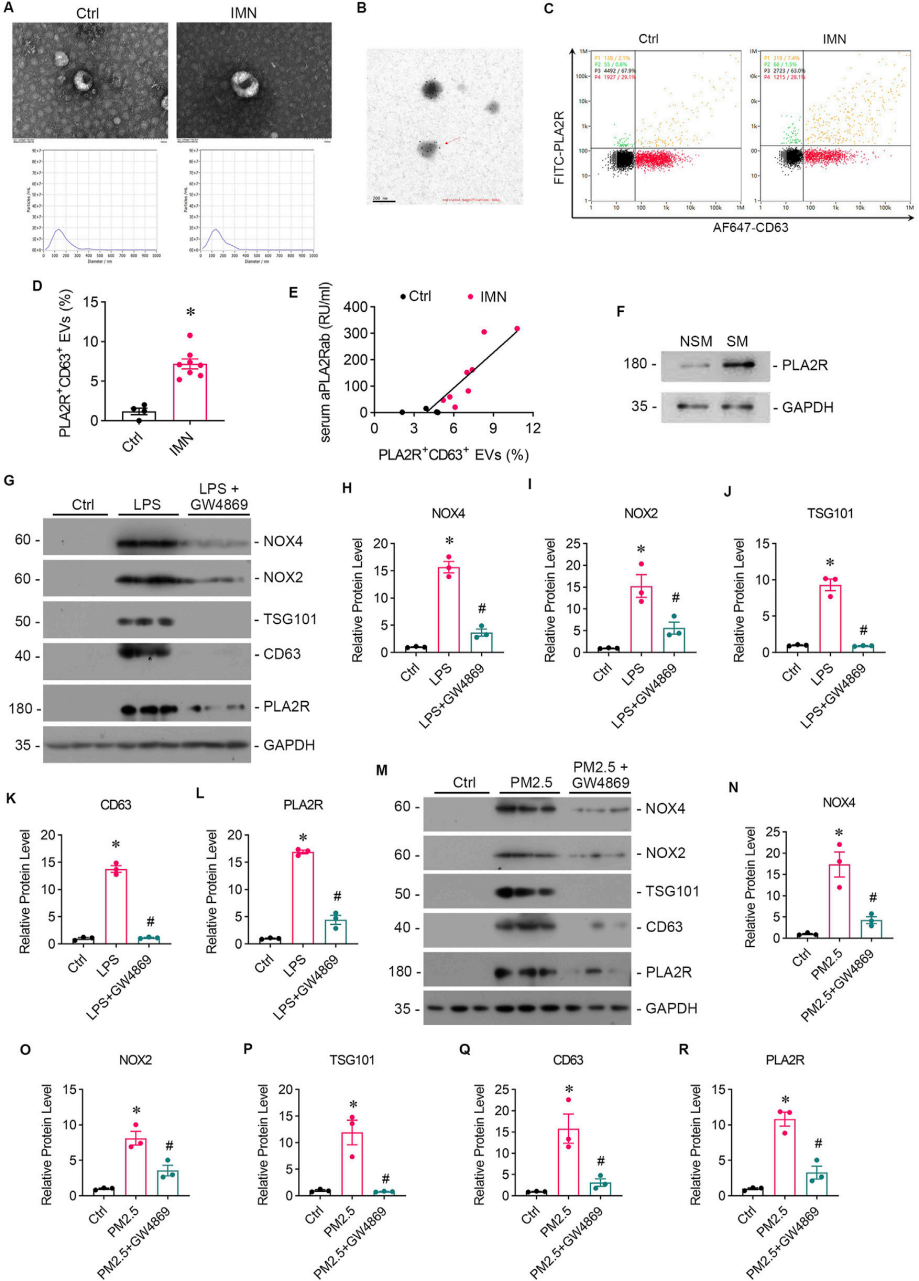
Patients with IMN exhibited increased serum levels of EVs, and oxidative stress enhanced EVs production *in vitro*. **(A)** TEM images displayed the morphology of EVs isolated from the serum of IMN patients. Scale bar = 100 nm. Patients with MCD served as controls. NTA images showed the distribution of EVs. **(B)** Representative images of immune colloidal gold electron microscopy demonstrated the presence of PLA2R on the surface of EVs, indicated by black dots. Scale bar = 200 nm. **(C, D)** Images from nano-flow cytometry and a corresponding statistical bar chart illustrated the proportion of PLA2R^+^ EVs in the serum of IMN patients compared to those with MCD. **(E)** A positive correlation was observed between the proportion of PLA2R + EVs and serum aPLA2Rab levels. **(F)** Representative Western blot analyses showed the PLA2R expression levels of EVs isolated from the lung tissue of smokers and non-smokers. **(G)** The levels of oxidative stress, PLA2R expression, and EVs production in Beas-2B cells were examined after stimulation with LPS, with or without GW4869 intervention. Representative Western blot **(G)** and quantitative data **(H–L)** are presented. The expression levels of NOX4 **(H)**, NOX2 **(I)**, TSG101 **(J)**, CD63 **(K)**, and PLA2R **(L)** were significantly upregulated by LPS, while GSH reversed this upregulation. (*p < 0.05 versus control; #*p* < 0.05 versus LPS). **(M–R)** The levels of oxidative stress, PLA2R expression, and EVs production in Beas-2B cells stimulated by RM8785 were analyzed, with or without GW4869 intervention. Representative Western blot **(M)** and quantitative data **(N–R)** are presented. The expression levels of NOX4 **(N)**, NOX2 **(O)**, TSG101 **(P)**, CD63 **(Q)**, and PLA2R **(R)** were significantly upregulated by RM8785, while GW4869 effectively reversed this upregulation. (*p < 0.05 versus control; #*p* < 0.05 versus PM2.5).

In our preliminary study, we employed immune colloidal gold electron microscopy and nano-flow cytometry to validate the presence of PLA2R^+^ EVs in the serum of patients diagnosed with IMN (Figures 4A, B). Our analysis revealed more PLA2R^+^ EVs in IMN patients than in MCD patients (Figures 4C, D). Notably, we identified a positive correlation between the proportion of PLA2R^+^ EVs and serum aPLA2Rab levels (Figure 4E). Furthermore, upon isolating EVs from the lung tissues of both smokers and non-smokers, we observed significantly elevated PLA2R expression on EVs in IMN patients compared to the control group (Figure 4F).

Subsequently, we collected supernatants from LPS-stimulated and RM8785-stimulated Beas-2B cells, and detected elevated levels of EV markers CD63 and TSG 101 (Figures 2A, E, F, 2H, L, M), which were downregulated by GSH. This suggests that PM2.5 promotes lung epithelial cells to produce EVs via oxidative stess.

### 3.5 GW4869 attenuates podocytes injury caused by PM2.5-exposed bronchial epithelial cells and downregulate PLA2R expression

**FIGURE 5:**
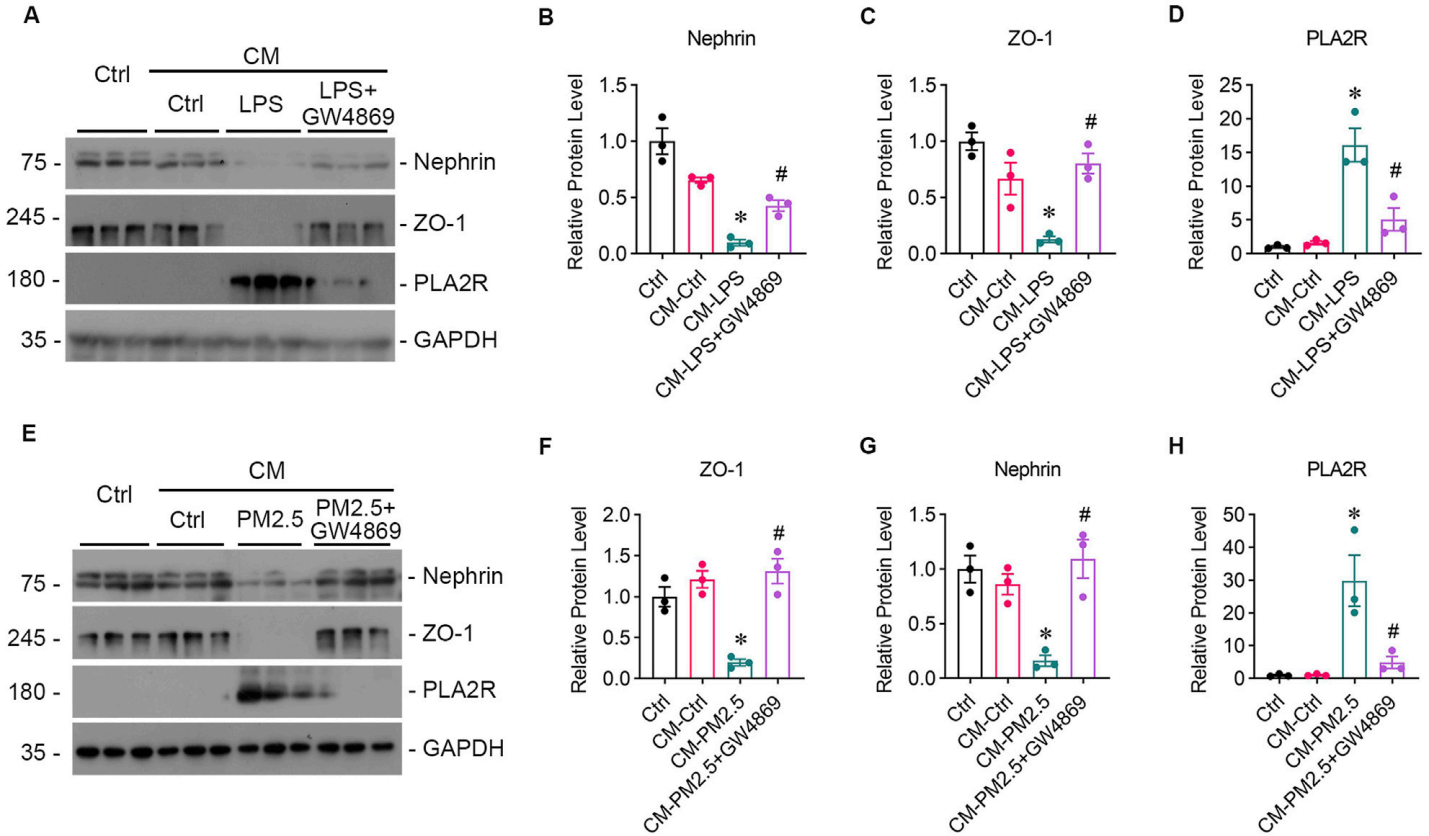
GW4869 alleviates podocyte injury induced by PM2.5-exposed bronchial epithelial cells. **(A)** Representative images from Western blot analyses illustrate Nephrin, ZO-1, and PLA2R protein expression levels in podocytes exposed to supernatants from LPS-stimulated Beas-2B cells, with and without GW4869 intervention. CM-LPS: conditioned media from Beas-2B cells stimulated by LPS. CM-LPS + GW4869: conditioned media from Beas-2B cells pretreated with GW4869 and then exposed to LPS. **(B, C)** The expression levels of Nephrin and ZO-1 were inhibited by CM-LPS but were partially restored by CM-LPS + GW4869. **(D)** The expression of PLA2R in podocytes significantly increased following exposure to CM-LPS. (*p < 0.05 versus CM-Ctrl; #*p* < 0.05 versus CM-LPS). **(E)** Western blot images show Nephrin, ZO-1, and PLA2R levels in podocytes exposed to supernatants from RM8785-stimulated Beas-2B cells, with and without GW4869 intervention. CM-PM2.5: conditioned media from RM8785-exposed Beas-2B cells. CM-LPS + GW4869: conditioned media from Beas-2B cells pretreated with GW4869 and then exposed to RM8785. **(F, G)** Nephrin and ZO-1 levels were inhibited by CM-PM2.5 but were partially elevated by CM-PM2.5 + GW4869. **(H)** PLA2R expression in podocytes increased with CM-PM2.5, and GW4869 mitigated this upregulation. (*p < 0.05 versus CM-Ctrl; #*p* < 0.05 versus CM-PM2.5).

GW4869, an inhibitor of EVs release, was found to reduce the oxidative stress and PLA2R expression level caused by LPS (Figures 4G–I, L) or PM2.5 (Figures 4M–O, R) in Beas-2B cells, or PM2.5 (Figures 4M–O, R) in Beas-2B cells, accompanied by barely expressed EVs markers (Figures 4J, K, P, Q), indicating a potential role of EVs in mediating oxidative stress. To investigate whether EVs mediate the crosstalk between podocyte and Beas-2B cells, Beas-2B cells were stimulated with LPS and pretreated with GW4869. The results demonstrated that GW4869 effectively attenuated the damage to podocytes caused by LPS-stimulated Beas-2B cells, which was evidenced by the recovery of nephrin (Figures 5A, B) and ZO-1 (Figures 5A, C) expression, as well as the reduction in PLA2R expression (Figures 5A, D). Subsequently, we explored the involvement of EVs in PM2.5-induced crosstalk between bronchial epithelial cells and podocytes. Treatment with GW4869 was found to significantly downregulate PLA2R expression and alleviate oxidative stress caused by PM2.5-exposed Beas-2B cells (Figures 5E–H).

**FIGURE 6:**
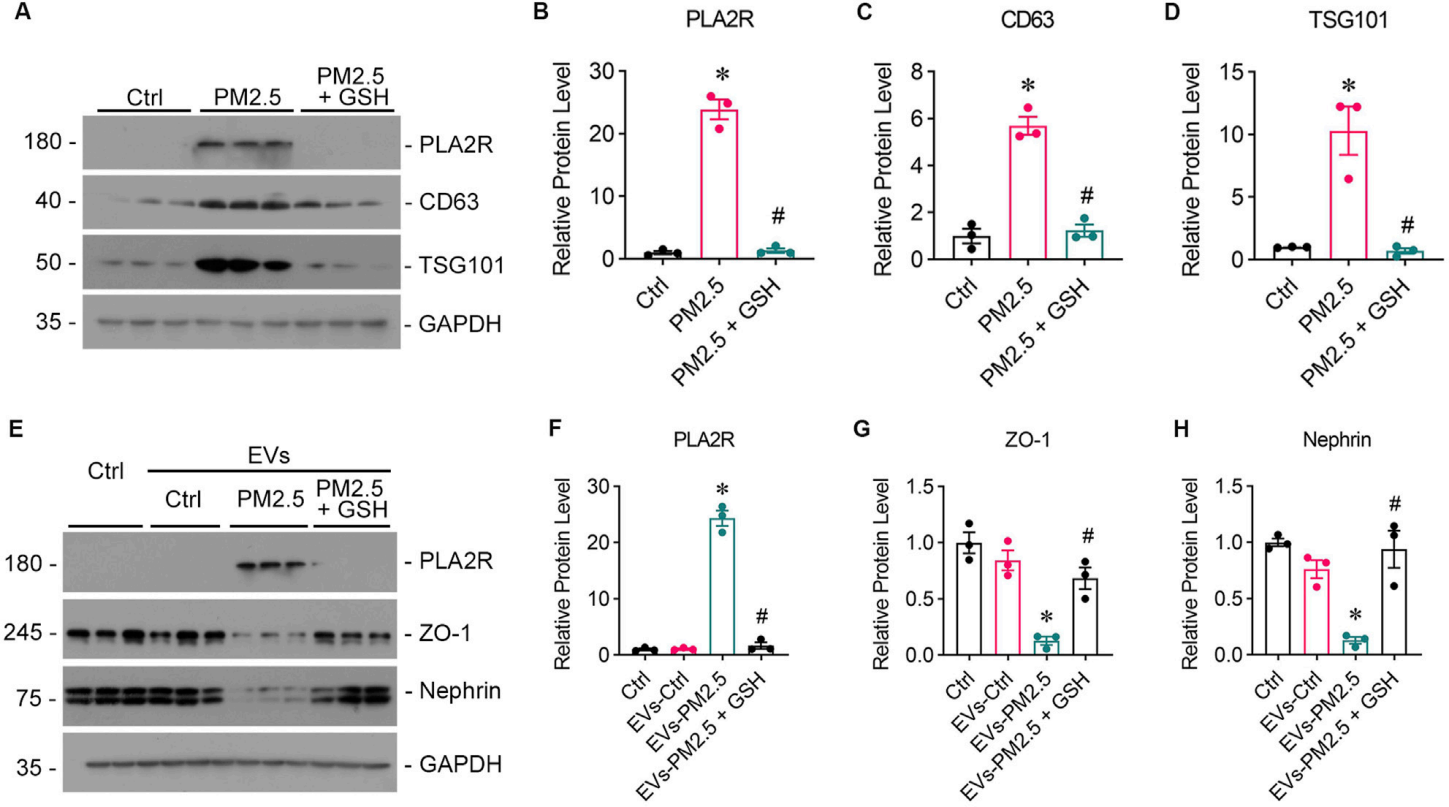
EVs from PM2.5-exposed Beas-2B cells induce podocyte injury, which can be mitigated by GSH. **(A)** Western blot analysis of EVs isolated from RM8785-exposed Beas-2B cells shows the expression of PLA2R, CD63, and TSG101. **(B–D)** PLA2R, CD63, and TSG101 expression levels were significantly higher in EVs from RM8785-exposed Beas-2B cells and were reduced by GSH pretreatment. (*p < 0.05 versus control; #*p* < 0.05 versus RM8785). **(E)** Western blot images depict Nephrin, ZO-1, and PLA2R expression levels in podocytes exposed to EVs from RM8785-stimulated Beas-2B cells, with or without GSH intervention. EVs-PM2.5: EVs from RM8785-exposed Beas-2B cells. EVs-PM2.5 + GSH: EVs from RM8785-exposed Beas-2B pretreated with GSH. **(F)** PLA2R expression in podocytes was increased by EVs-PM2.5 and significantly downregulated by GSH. **(G, H)** ZO-1 and Nephrin levels were decreased by EVs-PM2.5 and were partially restored by EVs-PM2.5 + GSH. (*p < 0.05 versus control; #*p* < 0.05 versus EVs-PM2.5).

To further validate the role of EVs in the communication between the lung and kidney, EVs were isolated from the supernatants of PM2.5-exposed Beas-2B cells via ultra-high-speed differential centrifugation, which was evidenced by Western blot analysis showing the high expression of CD63 (Figures 6A, C) and TSG101 (Figures 6A, D). The GSH could reduced the production of EVs and PLA2R expression on EVs from Beas-2B cells (Figure 6B). Subsequent treatment of podocytes with EVs secreted from PM2.5-exposed Beas-2B cells directly resulted in podocyte injury (Figures 6E, G, H) and upregulated PLA2R expression (Figures 6E, F).

## 4 Discussion

The present study provides new evidence that PM2.5 can stimulate PLA2R expression in the lung via oxidative stress. *In vitro* experiments have also revealed that extracellular vesicles mediate the PLA2R overexpression and podocyte injury caused by PM2.5-exposed lung epithelial cells.

In idiopathic MN, circulating autoantibodies target autoantigens which are expressed by podocytes, thus initiating the formation of immune complexes. It has been reported that patients with IMN exhibited recurrence rates of 30%–45% post-kidney transplantation (Debiec et al., 2011; Stahl et al., 2010), suggesting that extrarenal factors probably contribute to pathogenic autoantibody production. Burbelo et al. (2020) detected circulating PLA2R antibodies months to years before a biopsy-confirmed diagnosis of MN and in patients with non-nephrotic range proteinuria, indicating that podocyte-specific antibodies likely originate from extrarenal immune activation. Apart from being expressed in glomerular podocytes, PLA2R1 has been proven to get involved in several respiratory conditions such as asthma (Nolin et al., 2016) and COPD(Beaulieu et al., 2021) and overexpressed in bronchial epithelium, alveolar epithelium, lung macrophages, and vascular smooth muscle (Ancian et al., 1995; Granata et al., 2005; Silliman et al., 2002). We compared PLA2R expression in the lung tissues of smokers and non-smokers and detected enhanced PLA2R expression in the alveolar type Ⅱ epithelium of smokers. Based on the epidemiological studies implicating the association of PM2.5 (Li et al., 2018; Xu et al., 2016) or smoking (Mobarrez et al., 2014) and MN development, we speculated that PM2.5 stimulates PLA2R1 expression in the lung and promotes PLA2R antibody production in MN.

Previous literature confirms that oxidative stress is essential to PLA2R antigenic epitope exposure. The PLA2R molecule requires more than one disulfide bond in each of the three structural domains to undergo a conformational change that exposes the target antigenic epitope (Beck et al., 2009; Fresquet et al., 2015; Kao et al., 2015). When intracellular proteins are exposed to extracellular oxidative stress, disulfide bonds are formed to stabilize the spatial structure of the peptide chain. In order to ascertain the potential link between oxidative stress and PLA2R expression in lung tissue, bronchial epithelium was subjected to treatment with LPS and PM2.5, both well-known stimuli of oxidative stress. We observed a notable upregulation of PLA2R expression in response to the LPS and PM2.5 stimuli. Furthermore, we found that the antioxidant GSH effectively mitigated oxidative stress and alleviated the overexpression of PLA2R. These findings validate the role of oxidative stress in modulating PLA2R expression in lung tissue.

The adverse impact of PM2.5 on renal function encompasses a range of mechanisms, such as inflammation (Chenxu et al., 2018), oxidative stress (Xu et al., 2020; Xu et al., 2018), apoptosis (Zheng et al., 2020), DNA damage, (Jiang et al., 2020), and autophagy (Wang et al., 2021). In our exploration of the influence of PM2.5 exposure on the development of MN, we employed supernatants from bronchial epithelial cells exposed to PM2.5 to treat podocytes. Our findings revealed heightened PLA2R expression and podocyte injury, which underscore the potential interplay between lung and kidney in the context of PM2.5 exposure, warranting further investigation into the underlying mechanisms.

Extracellular vesicles are lipid bilayer membrane structures released by all cell types through various biogenesis pathways (Yanez-Mo et al., 2015). As crucial mediators of intercellular communication, exosomes transport proteins, DNAs, mRNAs, miRNAs, and more, playing essential roles in cellular homeostasis and mediating pathophysiological processes. Extensive research has elucidated the immunological impacts of EVs in antigen presentation, immune activation, suppression, and surveillance (Robbins and Morelli, 2014). Airborne fine particulate matter (Neri et al., 2016) and smoking can induce the production of exosomes (Baek et al., 2016; Li et al., 2010; Mobarrez et al., 2014) and inflammatory mediators via oxidative stress, involving redox-dependent modifications of thiol groups (Benedikter et al., 2018) and the stimulation of cellular membrane outgrowth by reactive carbonyl substances (RCS) and reactive oxygen species (ROS). Antioxidants such as N-acetylcysteine (NAC) and glutathione S-transferase (GSH) can concurrently inhibit the oxidation of cellular protein thiols and the release of exosomes (Benedikter et al., 2017; Benedikter et al., 2018). Our investigation revealed elevated levels of PLA2R^+^ EVs in the serum of patients with IMN and and from the lung tissue of smokers. *In vitro*, we observed that oxidative stress could induce bronchial epithelial cells to release more EVs. We isolated EVs from the supernatant of bronchial epithelial cells exposed to PM2.5. Intriguingly, EVs directly caused podocye injury and PLA2R overexpression, and the inhibition of EVs release from bronchial epithelial cells significantly reduced PLA2R expression in podocyte. On one hand, EVs can carry proteins, miRNA, and other substances that upregulate PLA2R expression, participating in the pathogenesis of MN. A search of the TargetScanHuman8.0 database (https://www.targetscan.org/vert_80/] revealed that differentially expressed genes, such as miR-30b-5p and miR-9-5p, in the urinary exosomes of IMN patients potentially regulate PLA2R1. The high-throughput sequencing results of urinary exosomal miRNA expression profiles in healthy controls and IMN patient demonstrated significant downregulation of miRNAs, including miR-532-3p, miR-9-5p, miR-30b-5p, miR-129-5p, miR-125b, and miR-338-5p, in IMN patients (Guo et al., 2022; Zhang et al., 2020). Besides, miR-30b-5p was found to negatively correlated with anti-PLA2R antibodies. On the other hand, PLA2R-positive exosomes may act as antigens, inducing immune cells to synthesize pathogenic antibodies.

The study was subject to several limitations. Firstly, rodents do not express PLA2R in the kidney. Thus, this study needs an animal model of membranous nephropathy to substantiate the results. Secondly, the specific molecular pathways of EVs-mediated podocyte injury still need to be clarified. Thirdly, the precise identification of the types of cells that produce EVs has yet to be determined. For instance, while macrophages are known to play a crucial role in PM2.5-induced lung inflammation, further investigation is required to ascertain whether EVs originating from macrophages participate in mediating crosstalk with podocytes.

## 5 Conclusion

Our findings demonstrate that PM2.5 triggers pulmonary PLA2R expression via oxidative stress. Furthermore, EVs derived from bronchial epithelium actively mediate the overexpression of PLA2R and subsequent podocyte injury. EVs play a pivotal role in mediating the crosstalk between the lung and kidney, highlighting the potential significance of EV-mediated signalling pathways in inter-organ communication and disease pathogenesis. These results strengthen our understanding of the mechanism underlying PM2.5 and MN development and provide new insights for therapeutic interventions aimed at MN prevention.

## Data Availability

The raw data supporting the conclusions of this article will be made available by the authors, without undue reservation.
